# Blood pressure percentiles by age and height for non-overweight Chinese children and adolescents: analysis of the china health and nutrition surveys 1991–2009

**DOI:** 10.1186/1471-2431-13-195

**Published:** 2013-11-25

**Authors:** Weili Yan, Fang Liu, Xuesong Li, Lin Wu, Yi Zhang, Yi Cheng, Wenhao Zhou, Guoying Huang

**Affiliations:** 1 Department of Clinical Epidemiology, Children’s Hospital of Fudan University, 399 Wanyuan Road, Shanghai 201102, China; 2 Cardiac Center, Children’s Hospital of Fudan University, Shanghai, P.R. China; 3 Department of Vascular Surgery, The No 5 Hospital of Shanghai, Shanghai, P.R. China; 4 Department of Neonatology, Children’s Hospital of Fudan University, Shanghai, P.R. China

**Keywords:** Adolescents, Blood pressure, Hypertension, Prevention, Reference

## Abstract

**Background:**

Hypertension is an important health problem in China and raised blood pressure in children may lead to future hypertension. Accordingly we aimed to provide a reference blood pressure table for age, gender and height in Chinese children.

**Methods:**

A reference sample of subjects was drawn from the Chinese Health and National Survey 1999–2009 aged 7–17 years after excluding overweight and obese children, the 50th, 90th and 95th percentiles of systolic and diastolic blood pressure (SBP and DBP)are presented corrected for height and age by gender. These values are compared with existing Chinese and US recommendations.

**Results:**

Results for the 50th, 90th and 95th percentile of SBP and DBP for 6245 boys and 5707 girls were presented by age and height percentiles. These observations were lower than existing Chinese recommendations before 13 years of age at median heightbut went higher in those >13 years old. At same age and height, SBP levels of American children were overall higher than Chinese counterparts from this study by average 9–10 mm Hg, but DBP did not show overall or significant difference.

**Conclusions:**

The first height-specific blood pressure reference values are proposed for Chinese children and adolescents aged 7–17 years. These are lower than existing US reference values and current Chinese cutoffs.

## Background

High blood pressure in children and adolescents is more common and is associated with increasing childhood obesity in western countries [1-3] as well as in China [4,5]. Prehypertension and hypertension in childhood are associated with a 2.5 fold increase likelihood of adult hypertension [6,7]. In addition, childhood hypertension correlates with early atherosclerosis, impaired arterial compliance [8-10], cardiac structural changes [11], and additional risk factors for metabolic syndrome. In a recent longitudinal study of 342 children aged 11–15 years, childhood BP was found to predict early adulthood dyslipidaemia, independently of body mass index (BMI) [12]. For the pediatric population, the percentile of blood pressure is used since blood pressure changes with age and body size. The 90th and 95th percentiles of blood pressure by age and height are recommended by the fourth report on the diagnosis, evaluation and treatment of high blood pressure in children and adolescents to define prehypertension and hypertension respectively [13].

National blood pressure cutoffs by age groups for Chinese children were first published in 1992, and updated in 2010 [14], however, height was not taken into account in either of them. In order to achieve more accurate blood pressure evaluation in children, the aim of this study is to establish mercury blood pressure reference values by age and height Chinese children and adolescents aged 7–17 years based on the nationally representative study sample, China Health and Nutrition Survey (CHNS).

## Methods

We used data from the China Health and Nutrition Survey (CHNS) [15-17]. General information, methods and dataset information can be accessed from the website (http://www.cpc.unc.edu/projects/china) [15-17]. Briefly, participants were sampled from seven Chinese provinces (namely Jiangsu, Shandong, Henan, Hubei, Human, Guangxi and Guizhou). The survey design and methods have been described in detail elsewhere previously [16,17]. After exclusion of overweight and obese students based on the Chinese recommendation [18], boys and girls aged 7–17 years were included in this analysis. The University of North Carolina and the China Center for Disease Control and Prevention had reviewed and approved the procedures for data collection and all subjects and/or their parents/guardians have provided written informed consent.

### Measurements and definitions

Blood pressure was measured using mercury sphygmomanometer according to the standard protocol by trained and qualified observers, which was described elsewhere [5]. Korotkoff phase 1 and Korotkoff phase 5 were used for defining systolic blood pressure (SBP) and diastolic blood pressure (DBP). Appropriate size of cuff was used to measure blood pressure for children using the right arm. The mean of 2 measurements was analyzed. Height and weight were measured to calculate body mass index (BMI, weight in kilograms divided by the square of height in meters). Overweight and obese were defined according to the age- and gender-specific BMI reference standard for Chinese children and adolescents [18].

### Statistical methods

Percentiles of height as a function of age were obtained by smoothed centile curves modeled using LMS method [19] with the program LMSChartMaker Pro 2.3. The reference curves of blood pressure by age and height were simultaneously fitted by using an extension of the LMS method [19], namely the generalized additive model for location scale and shape (GAMLSS) with the Box-Cox power exponential (BCPE) distribution family or BOX-COC-t, fitted with GAMLSS 4.1-5 in the free statistical software R2.15.0 (http://www.cran.r-project.org/). GAMLSS is a generalization of the LMS method where Y has a specified frequency distribution D (μ,σ,ν,τ), the 4 parameters define the location, scale and shape of the blood pressure distribution with age and height. Linear effect and additive effect of age and height (two covariates) on SBP and DBP were modeled simultaneously to obtain the optimal models by minimizing the Schwarz Bayesian Criterion (SBC). The all possible functions of age and height as well as the interactions of which were considered in the modeling, the most fitted model were achieved. The reference values of 50th, 90th, and 95th percentiles of SBP and DBP were computed by age and height (exact heights according to the 5th, 25th, 50th, 75th and 95th percentiles) for boys and girls respectively.

To make comparisons with the existing Chinese blood pressure recommendations for children [14], heights and BMI were standardized according to the same reference populations, the Chinese National Survey on Constitution and Health (CNSCH 2005) [20]. Since height percentiles were not considered in the existing recommendation [14], blood pressure reference values with median height of this study were used to make comparisons.

Differences in proposed SBP and DBP cutoff values (the 50th, 90th and 95th percentiles) for Chinese boys and girls were compared with the Fourth Report on the Diagnosis, Evaluation, and Treatment of High Blood Pressure in Children and Adolescents of the US [13] at given age (7–17 years) and the median height (cm). Since the height distributions of the two reference samples are not comparable, it is difficult to compare the corresponding height–depended 50th, 90th and 95th blood pressure percentiles directly, therefore, the expected SBP and DBP of US counterparts with the median height of Chinese reference population were computed based on the equations. For example, the 50th, 90th and 95th percentiles of SBP and DBP for an US boy aged 7 with the median height of 120 cm, were calculated as 97, 111, 118 mm Hg as well as 57, 72, and 80 mm Hg, based on the US equation [13] presented in the table B1 of the Fourth report [13]. Stata 11.0 (StataCorp LP, StataCorp, Texas 77845 USA) were used for conventional descriptive analysis.

Independent software called “Blood Pressure Calculator “has been developed based on the fitted models of blood pressure from this study.

## Results

Based on Chinese national BMI cutoff points, a total of 620 boys and 445 girls (8.2%) were excluded from the original study sample (n = 13014) because of being overweight or obese. The remaining reference sample consists of children and adolescents aged 7–17 years (including 6245 boys and 5704 girls) with complete data on age, gender, height and three SBP and DBP readings. The characteristics of subjects were given in Table 1. It shows that the mean body weight, height, BMI, SBP and DBP increase with the age groups, the ranges of height for the three age groups vary from 50.5 cm to 58.4 cm.

**Table 1:** Baseline characteristics of the reference population of nonoverweight

	**Age, yrs**		
	**7-10**	**11-13**	**14-17**
Children excluded because of overweight/obese, n			
Boys	127	138	128
Girls	134	156	127
Children included, n			
Boys	1755	2775	2294
Girls	1656	2517	2074
Weight, kg			
Boys	25.0 ± 5.3	35.7 ± 9.1	51.2 ± 9.6
Girls	24.1 ± 5.1	35.6 ± 8.5	47.4 ± 7.2
Height, cm			
Boys	125.1 ± 8.4	143.0 ± 11.1	163.3 ± 8.9
Girls	124.1 ± 8.8	143.7 ± 10.2	155.8 ± 6.8
Height range, cm			
Boys	101.8-153.0	96.0-178.0	130.6-189.0
Girls	98.0-152.3	115.0-171.5	130.0-180.5
BMI, kg/m^2^			
Boys	15.2 ± 1.3	16.6 ± 1.8	18.9 ± 2.0
Girls	14.9 ± 1.3	16.6 ± 2.0	19.1 ± 2.0
SBP, mm Hg			
Boys	90.7 ± 11.9	96.4 ± 11.7	106.7 ± 12.4
Girls	89.8 ± 11.4	96.6 ± 12.0	104.0 ± 10.7
DBP, mm Hg			
Boys	59.7 ± 9.4	63.4 ± 8.8	69.4 ± 9.3
Girls	58.9 ± 9.6	63.4 ± 8.9	68.6 ± 8.1

Children and Adolescents (6245 boys and 5704 girls aged 7–17 yrs).

The optimal models for the 4 parameters of SBP and DBP distribution for boys and girls were fitted. It showed that BCPE model was the best fitted model for SBP for boys and DBP for both genders, BCT model was the best fitted model for SBP of girls. Reference values of the 50th, 90th, and 95th percentiles of SBP and DBP, were computed by age and exact heights according to the 5th, 25th, 50th, 75th and 95th percentiles ) for boys and girls respectively and presented as Table 2 (for boys) and Table 3 (for girls). At adolescents aged 17 years old with the median height (167.5 cm for boys and 157.8 cm for girls), the median SBP were 107 for boys and 105 for girls; the 90th percentiles of SBP and DBP were 122 mm Hg and 80 mm Hg for boys and 118 mm Hg and 79 mm Hg for girls respectively, which were very close to the cutoff of 120 /80 mm Hg for identifying prehypertension for all ages recommended by the fourth report [13]. The 95th percentile of SBP and DBP were 126 mm Hg and 83 mm Hg for boys, and 122 mm Hg and 82 mm Hg for girls respectively, which are lower than the recommended optimal blood pressure of 130/85 mm Hg for adults [21].

**Table 2:** Age-height-specific references: median, the 90th and 95th percentiles of SBP and DBP values for boys aged 7–17 years

**Age, years**	**Height, cm**	**Percentiles of height**	**SBP percentiles, mm Hg**				**DBP, mm Hg**			
			s*	**50th**	**90th**	**95th**	s*	**50th**	**90th**	**95th**
7	109.0	5th	0.13	82	96	101	0.16	55	67	70
7	115.0	25th	0.12	84	99	104	0.16	56	68	71
7	120.0	50th	0.12	86	101	106	0.16	57	69	72
7	125.0	75th	0.12	88	103	108	0.15	59	70	74
7	130.0	95th	0.12	90	105	110	0.15	60	71	75
8	113.7	5th	0.12	84	99	103	0.16	56	68	71
8	119.5	25th	0.12	86	101	105	0.15	58	69	72
8	124.5	50th	0.12	88	103	108	0.15	59	70	73
8	128.2	75th	0.12	90	105	109	0.15	60	71	74
8	135.4	95th	0.12	92	107	112	0.15	61	73	76
9	119.5	5th	0.12	87	101	106	0.15	58	69	72
9	125.4	25th	0.12	89	104	108	0.15	59	70	74
9	130.0	50th	0.12	91	105	110	0.15	60	72	75
9	135.1	75th	0.12	93	107	112	0.15	61	73	76
9	143.0	95th	0.11	96	111	115	0.14	63	75	78
10	122.0	5th	0.12	88	102	107	0.15	58	70	73
10	129.0	25th	0.12	91	105	110	0.15	60	71	75
10	134.0	50th	0.12	92	107	112	0.14	61	72	76
10	140.0	75th	0.11	95	110	114	0.14	62	74	77
10	146.4	95th	0.11	97	112	117	0.14	64	75	79
11	126.7	5th	0.12	90	104	109	0.15	60	71	74
11	134.0	25th	0.12	93	107	112	0.14	61	72	76
11	139.3	50th	0.11	95	109	114	0.14	62	74	77
11	144.6	75th	0.11	97	112	116	0.14	64	75	78
11	153.0	95th	0.11	100	115	119	0.13	66	77	80
12	133.0	5th	0.11	93	107	111	0.14	61	72	75
12	139.5	25th	0.11	95	110	114	0.14	63	74	77
12	145.5	50th	0.11	97	112	116	0.13	64	75	78
12	152.0	75th	0.11	100	115	119	0.13	66	77	80
12	162.0	95th	0.11	104	119	123	0.13	68	79	82
13	135.0	5th	0.11	94	108	112	0.14	62	73	76
13	145.0	25th	0.11	98	112	116	0.13	64	75	78
13	152.0	50th	0.11	100	115	119	0.13	66	77	80
13	158.1	75th	0.11	103	117	122	0.13	67	78	81
13	168.1	95th	0.10	106	121	126	0.12	69	80	84
14	142.0	5th	0.11	97	111	115	0.13	64	74	78
14	152.0	25th	0.11	101	115	119	0.13	66	77	80
14	159.5	50th	0.11	103	118	122	0.13	68	78	82
14	165.0	75th	0.10	106	120	125	0.12	69	80	83
14	172.5	95th	0.10	108	123	128	0.12	70	81	85
15	147.9	5th	0.11	99	114	118	0.13	65	76	79
15	157.0	25th	0.11	103	117	121	0.12	67	78	81
15	163.0	50th	0.10	105	120	124	0.12	68	79	82
15	168.3	75th	0.10	107	122	126	0.12	70	80	84
15	175.0	95th	0.10	110	124	129	0.12	71	82	85
16	153.0	5th	0.11	102	116	120	0.12	66	77	80
16	160.5	25th	0.10	104	119	123	0.12	68	79	82
16	165.0	50th	0.10	106	121	125	0.12	69	80	83
16	170.0	75th	0.10	108	123	127	0.12	70	81	84
16	177.0	95th	0.10	111	125	130	0.12	72	83	86
17	155.0	5th	0.10	103	117	121	0.12	67	78	80
17	163.0	25th	0.10	106	120	124	0.12	69	79	82
17	167.5	50th	0.10	107	122	126	0.12	70	80	83
17	172.0	75th	0.10	109	124	128	0.12	71	81	84
17	180.0	95th	0.10	112	127	131	0.11	73	83	86

Notes

SBP, systolic blood pressure; DBP, diastolic blood pressure.

*s, the coefficient of variation of blood pressure.

**Table 3:** Age-height-specific references: median, the 90th and 95th percentiles of SBP and DBP values for girls aged 7–17 years

**Age, years**	**Height, cm**	**Percentile of height**	**SBP, mm Hg**				**DBP, mm Hg**			
			s*	**50th**	**90th**	**95th**	s*	**50th**	**90th**	**95th**
7	108.0	5th	0.12	82	96	100	0.14	54	64	67
7	114.2	25th	0.12	84	98	103	0.14	56	66	69
7	118.2	50th	0.12	86	100	104	0.14	56	67	70
7	122.0	75th	0.12	87	101	105	0.14	57	68	71
7	129.6	95th	0.12	89	103	108	0.14	59	70	73
8	113.2	5th	0.12	85	99	103	0.14	56	66	69
8	119.3	25th	0.12	87	101	105	0.14	57	67	70
8	123.0	50th	0.12	88	102	106	0.14	58	68	71
8	128.5	75th	0.12	89	104	108	0.14	59	70	72
8	135.0	95th	0.11	91	106	110	0.14	60	71	74
9	119.0	5th	0.12	87	101	105	0.14	57	68	70
9	124.5	25th	0.12	89	103	107	0.14	59	69	72
9	130.0	50th	0.11	91	105	109	0.14	60	70	73
9	134.8	75th	0.11	92	106	110	0.14	61	71	74
9	143.0	95th	0.11	95	109	113	0.14	62	73	76
10	123.0	5th	0.12	89	103	107	0.13	59	69	72
10	219.6	25th	0.09	119	133	137	0.13	78	92	95
10	135.2	50th	0.11	93	107	111	0.13	61	72	75
10	140.0	75th	0.11	94	108	112	0.13	62	73	76
10	148.0	95th	0.11	97	111	115	0.13	64	75	78
11	128.0	5th	0.11	91	105	109	0.13	60	70	73
11	135.0	25th	0.11	94	107	111	0.13	62	72	75
11	141.1	50th	0.11	95	109	113	0.13	63	73	76
11	147.7	75th	0.11	97	111	115	0.13	64	75	78
11	156.0	95th	0.10	100	114	118	0.13	66	77	80
12	132.6	5th	0.11	94	107	111	0.12	62	72	74
12	142.0	25th	0.11	96	110	114	0.12	63	74	77
12	147.0	50th	0.11	98	112	116	0.12	64	75	78
12	153.0	75th	0.10	100	114	118	0.12	66	76	79
12	159.4	95th	0.10	102	116	120	0.12	67	78	81
13	138.2	5th	0.11	96	109	113	0.12	63	73	76
13	146.3	25th	0.10	98	112	116	0.12	65	75	78
13	151.2	50th	0.10	100	114	118	0.12	66	76	79
13	156.1	75th	0.10	101	115	119	0.12	67	77	80
13	162.0	95th	0.10	103	117	121	0.12	68	79	82
14	143.0	5th	0.10	98	112	116	0.12	65	75	77
14	150.0	25th	0.10	100	114	118	0.12	66	76	79
14	154.2	50th	0.10	102	115	119	0.12	67	77	80
14	158.9	75th	0.10	103	117	121	0.12	68	78	81
14	165.0	95th	0.10	105	118	122	0.12	69	80	83
15	144.2	5th	0.10	99	113	116	0.11	65	75	78
15	151.0	25th	0.10	101	115	119	0.11	67	77	80
15	155.6	50th	0.10	103	116	120	0.11	68	78	81
15	160.0	75th	0.10	104	117	121	0.11	68	79	82
15	165.8	95th	0.10	106	119	123	0.11	70	80	83
16	145.8	5th	0.10	100	114	118	0.11	66	76	79
16	152.9	25th	0.10	102	116	120	0.11	67	77	80
16	157.0	50th	0.10	104	117	121	0.11	68	78	81
16	161.0	75th	0.10	105	118	122	0.11	69	79	82
16	167.6	95th	0.09	107	120	124	0.11	70	81	84
17	145.6	5th	0.10	101	114	118	0.11	66	76	79
17	153.1	25th	0.10	103	116	120	0.11	68	78	80
17	157.8	50th	0.09	105	118	122	0.11	69	79	82
17	162.4	75th	0.09	106	119	123	0.11	70	80	83
17	168.0	95th	0.09	108	121	125	0.11	71	81	84

Notes

SBP, systolic blood pressure; DBP, diastolic blood pressure.

*s, the coefficient of variation of blood pressure.

### Comparison with available Chinese reference blood pressure tables

**Figure 1:**
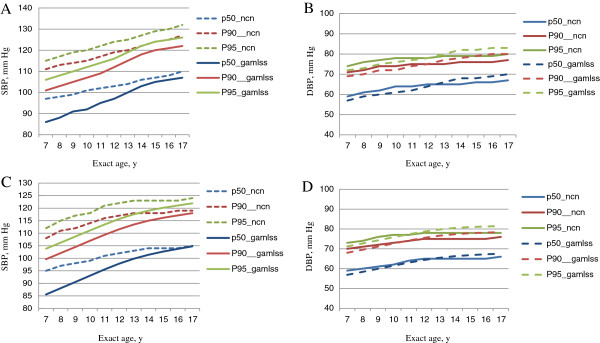
**The 50th, 90th and 95th percentile of SBP and DBP for non-overweight Chinese (CHNS) with the median height compared with the national recommendation **[14]**. A**,SBP for boys; **B**, DBP for boys; **C**, SBP for girls; **D**, DBP for girls. Pn_ncn indicates the existing national centile curves, Pn_gamlss indicates proposed centile curves by the current study.

**Figure 2:**
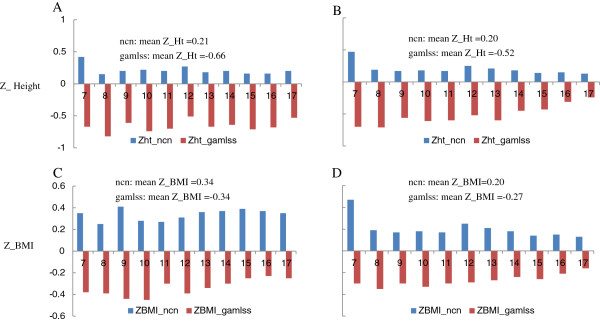
**Standardized BMI(Z_BMI) and height (Z-Ht) of the reference study sample from the current study and the earlier national recommendation.** The standardized height (Z-Ht) and BMI (Z_BMI) of reference sample of the current study was compared with that from the national recommendation [14], showing that the reference sample of current study is shorter (0.88 SD for boys and 0.70 SD for girls) and thinner (0.68 SD for boys and 0.47 SD for girls) compared with the reference sample used by national recommendation [14]. **A**, Z_height for boys; **B**, Z_height for girls; **C**, Z_BMI for boys; **D**, Z_BMI for girls.

Figure 1 showed the 90th percentiles of SBP and DBP for boys and girls aged 7–17 years by the 5th, 50th and 95th percentiles of height compared with that from the study by Mi J et al. [14]. It showed that SBP values for children at all ages with the median height proposed by this study were 5–10 mm Hg lower. DBP was 2–3 mm Hg higher before age of 14, but tended to be similar afterwards. As shown in Figure 2, the reference sample of current study is shorter (0.88 SD for boys and 0.7 SD for girls) and thinner (0.68 SD for boys and 0.47 SD for girls) compared with the reference sample used by national recommendation [14] than the reference sample used the earlier study [14].

Height is not taken into account in the earlier national recommendation. The 50th, 90th and 95th centile curves of SBP proposed by the current study were lower than the existing national non-height specific reference, the differences tended to decrease after 14 years old. However, the three centile curves of the current study were lower than then existing national reference curves before age of 11–12 years but exceeded it up to 17 years old to the similar extent with the age of 7. This trend remained similar in boys and girls.

### Comparison with US national reference tables

**Figure 3:**
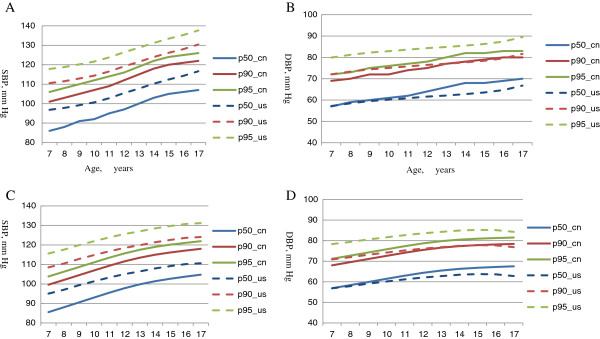
**The 50th, 90th and 95th percentile of SBP and DBP for the median height for Chinese (CHNS) and American boys (A) and girls (B).** SBP and DBP values for American children with the given age (years) and height (cm) were computed based on SBP and DBP equations from the table B1 of the Fourth Report [13], without exclusion of overweight or obese children. It shows that American boys and girls at given age and height (cm) according to Chinese children had higher SBP and DBP percentile levels (the 50th, 90th and 95th ). **A**, SBP for boys; **B**, DBP for boys; **C**, SBP for girls; **D**, DBP for girls. Pn _ncn indicates the existing national centile curves, Pn_us indicates corresponding expected centile curves of American children based on equations in Table B1 in [13].

Compared with the international blood pressure table recommended by the Fourth report [13] (Figure 3), the 50th, 90th and 95th percentiles of SBP in Chinese boys and girls were averagely 9–10 mm Hg lower than the expected values for the American counterparts; however, there were no clear difference in DBP percentiles.

## Discussion

We present the first height percentile specific-blood pressure references in China and compare these with the earlier ones also international values. These new cutoffs will be more accurate for evaluating blood pressure levels for children and adolescents with extreme heights. The proposed 90th and 95th percentiles of blood pressure may be used to detect prehypertension and hypertension in Chinese pediatric population. The using of non-overweight reference sample may makes the proposed blood pressure cutoff points more sensitive to identify children with elevated blood pressure because of with risk factors such as being overweight or obese. The 99th percentiles are not proposed based on the thinking that children with blood pressure measurements over it will not be directly diagnosed as hypertension, instead, additional blood pressure measurements will be suggested.

Height is a key covariate associated with blood pressure levels. The ignoring of height of the blood pressure references may result in inaccurate blood pressure evaluation in pediatric practice especially for children who are very short or tall. Since there may be significant difference in height distribution between the current study sample and others, the blood pressure cutoffs for exact height values instead of height percentile categories are proposed to make it more practical and accurate for individual blood pressure assessment.

Compared with the existing national age-specific blood pressure recommendations [14], the blood pressure percentiles for age proposed by the current study were lower. In the earlier recommendations [14], only function of age on blood pressure was considered, the blood pressure percentiles could be understand as the functions of age and average height of the study population. It may be appropriate for those with average height, while it may make inaccurate estimation of blood pressure for children with extreme heights. The current study uses new statistical method GAMLSS model, which is able to handle two and more covariates to allowance to fit functions of both age and height to blood pressure levels. Compared with the earlier recommendation [14], the percentiles proposed by the current study will be more accurate especially for those children with extreme heights. In addition, exclusion of overweight and obese subjects from the reference population of the current study makes lower BMI levels compared with that used by the earlier study (BMI Z-score difference is averagely 0.68SD for boys and 0.47SD for girls). The corresponding lower cutoffs of blood pressure we propose are expected to be more sensitive to identify obesity-related high blood pressure in children.

In overall, the blood pressure percentiles we proposed are lower than the international one for American children [13], which also consider both functions of age and height on blood pressure levels. The racial difference of blood pressure reference values for given age and height supports the necessity of establishing blood pressure references for Chinese children and adolescents, in order to achieve early prevention of childhood hypertension in the country.

Given the complex calculations for individual assessment of blood pressure in practice, a Blood Pressure Calculator has been developed based on the fitted models of blood pressure from this study. After inputting age, gender, height, SBP and DBP levels, it will return SBP and DBP percentiles and blood pressure status (normotensive, prehyepertensive or hypertensive). This calculator may greatly help individual clinical evaluation of blood pressure in hospitals and public health settings.

Our study has some limitations. The sample size of boys and girls aged 7–17 years are relatively small compared with the total population of China, and survey fields cover only 7 east coast provinces. However, we choose to use CHNS data is based on the considerations that the CHNS study are jointly funded by Chinese government and American organizations, the methodology of blood pressure measurement, quality control and data management follow international criteria, the international comparisons will be more convincing. Second, no external validation was made to assess the performance of the newly proposed blood pressure tables. A second validation study would be helpful to compare the accuracy of age- and height-specific blood pressure percentiles from nonoverweight reference sample proposed by the current study with the existing age-specific percentiles but without excluding overweight subjects [14] in children’s blood pressure assessment.

## Conclusion

In summary, the current study proposes the first age and height corrected blood pressure percentiles for Chinese children and adolescents aged 7–17 years with potential for more accurate blood pressure evaluation for children with extreme height, and in identifying obesity-related high blood pressure. It is expected that the proposed new references will be used in clinical individual blood pressure evaluation and government-supported annual national regular school-based fitness and physical survey in China.

## Competing interests

The authors declare no financial or non-financial competing interests.

## Authors’ contributions

AB carried out the molecular genetic studies, participated in the sequence alignment and drafted the manuscript. JY carried out the immunoassays. MT participated in the sequence alignment. ES participated in the design of the study and performed the statistical analysis. FG conceived of the study and participated in its design and coordination and helped to draft the manuscript. All authors read and approved the final manuscript. WY conceptualized and designed the study, supervised the gamlss modeling and all the statistical analyses, drafted the manuscript, and approved the final manuscript as submitted. FL, XL, LW, YZ and YC carried out the conventional statistical analyses, made the tables, reviewed and revised the manuscript, and approved the final manuscript as submitted. WZ and GH participated design, data interpretation, discussion and approved the final manuscript as submitted.

## Pre-publication history

The pre-publication history for this paper can be accessed here:

http://www.biomedcentral.com/1471-2431/13/195/prepub

## References

[B1] McNiece KL Poffenbarger TS Turner JL Franco KD Sorof JM Portman RJ Prevalence of hypertension and pre-hypertension among adolescents J Pediatr 2007 150 6 640 644 644 e641 10.1016/j.jpeds.2007.01.052 17517252

[B2] Chiolero A Cachat F Burnier M Paccaud F Bovet P Prevalence of hypertension in schoolchildren based on repeated measurements and association with overweight J Hypertens 2007 25 11 2209 2217 10.1097/HJH.0b013e3282ef48b2 17921814

[B3] Chiolero A Paccaud F Bovet P Pre-hypertension and hypertension among adolescents of Switzerland J Pediatr 2007 151 6 e24 e25 10.1016/j.jpeds.2007.08.043 18035125

[B4] Cui Z Huxley R Wu Y Dibley MJ Temporal trends in overweight and obesity of children and adolescents from nine Provinces in China from 1991–2006 Int J Pediatr Obes 2010 5 5 365 374 10.3109/17477166.2010.490262 20836722

[B5] Liang YJ Xi B Hu YH Wang C Liu JT Yan YK Xu T Wang RQ Trends in blood pressure and hypertension among Chinese children and adolescents: China Health and Nutrition Surveys 1991–2004 Blood Press 2011 20 1 45 53 10.3109/08037051.2010.524085 21047169

[B6] Chen X Wang Y Tracking of blood pressure from childhood to adulthood: a systematic review and meta-regression analysis Circulation 2008 117 25 3171 3180 10.1161/CIRCULATIONAHA.107.730366 18559702 PMC3568631

[B7] Lauer RM Clarke WR Childhood risk factors for high adult blood pressure: the Muscatine Study Pediatrics 1989 84 4 633 641 2780125

[B8] Mahoney LT Burns TL Stanford W Thompson BH Witt JD Rost CA Lauer RM Coronary risk factors measured in childhood and young adult life are associated with coronary artery calcification in young adults: the Muscatine Study J Am Coll Cardiol 1996 27 2 277 284 8557894 10.1016/0735-1097(95)00461-0

[B9] Berenson GS Srinivasan SR Bao W Newman WP 3rd Tracy RE Wattigney WA Association between multiple cardiovascular risk factors and atherosclerosis in children and young adults. The Bogalusa Heart Study N Engl J Med 1998 338 23 1650 1656 10.1056/NEJM199806043382302 9614255

[B10] Lande MB Carson NL Roy J Meagher CC Effects of childhood primary hypertension on carotid intima media thickness: a matched controlled study Hypertension 2006 48 1 40 44 10.1161/01.HYP.0000227029.10536.e8 16735644

[B11] Zhu H Yan W Ge D Treiber FA Harshfield GA Kapuku G Snieder H Dong Y Cardiovascular characteristics in American youth with pre-hypertension Am J Hypertens 2007 20 10 1051 1057 10.1016/j.amjhyper.2007.05.009 17903687

[B12] Rademacher ER Jacobs DR Jr Moran A Steinberger J Prineas RJ Sinaiko A Relation of blood pressure and body mass index during childhood to cardiovascular risk factor levels in young adults J Hypertens 2009 27 9 1766 1774 10.1097/HJH.0b013e32832e8cfa 19633567 PMC2886129

[B13] National High Blood Pressure Education Program Working Group on Hypertension Control in Children and Adolescents The fourth report on the diagnosis, evaluation, and treatment of high blood pressure in children and adolescents Pediatrics 2004 114 2 Suppl 4th Report 555 576 15286277

[B14] Mi J Wang T Meng L Zhu G Han S Zhong Y Liu G Wan Y Xiong F Shi J Development of blood pressure reference standards for Chinese children and adolescents Chin J Evid Based Pediatr 2010 5 1 4 14

[B15] CHNS China health and nutrition survey: design and methods http://www.cpc.unc.edu/projects/china/about/design

[B16] Liu H The China health and nutrition survey: an important database for poverty and inequality research J Econ Inequal 2008 6 373 376 10.1007/s10888-008-9088-x

[B17] Popkin BM Du S Zhai F Zhang B Cohort Profile: the China Health and Nutrition Survey–monitoring and understanding socio-economic and health change in China, 1989–2011 Int J Epidemiol 2010 39 6 1435 1440 10.1093/ije/dyp322 19887509 PMC2992625

[B18] Ji CY Report on childhood obesity in China (1)–body mass index reference for screening overweight and obesity in Chinese school-age children Biomed Environ Sci 2005 18 6 390 400 16544521

[B19] Cole TJ Green PJ Smoothing reference centile curves: the LMS method and penalized likelihood Stat Med 1992 11 10 1305 1319 10.1002/sim.4780111005 1518992

[B20] Work Group of Physical Fitness and Health Surveillance of Chinese School Students Report on the Physical Fitness and Health Surveillance of Chinese School Students Chinese high education press ISBN: 9787040212006)*.*2007(1^st^ ed)

[B21] The IDF consensus worldwide definition of the metabolic syndromehttp://www.idf.org/metabolic-syndrome

